# Cortical perforation combined with Masquelet technique to treat extensive bone and soft tissue injury: A case report

**DOI:** 10.1097/MD.0000000000035468

**Published:** 2023-10-13

**Authors:** Yingying Deng, Zhijun Dong, Yuan Pan, Congtao Wang, Min An, Maohua Tang, Fuyao Liu

**Affiliations:** a Department of Lower Limb Trauma, Beijing Jishuitan Hospital, Guizhou Hospital, Baiyun District, Guiyang, Guizhou, China.

**Keywords:** cortical perforation, Masquelet technique, bone and soft tissue injury, bone cement

## Abstract

**Introduction:**

We report a case of a serious traffic accident injury to the lower leg involving a large skin defect with the long bone exposed. In this situation, the usual intervention is flap transplantation after debridement and infection control by completely covering the wound. Flap transplantation has certain limitations; therefore, we chose the surgical strategy of cortical bone drilling-induced membrane technology (Masquelet technique).

**Case presentation:**

A 28-year-old healthy man was injured in a car accident and presented to the local hospital with a large skin defect and exposed left lower leg long bone. After transfer to our hospital, the patient underwent repeated debridement and skin graft, a cortex borehole combined with bone cement cover, and ankle fusion. The patient achieved full recovery.

**Conclusion:**

From our experience in treating this case, we conclude that large skin defects, periosteal stripping, and bone exposure due to physical injury can be successfully treated with cortical perforation and the Masquelet technique so as to avoid flap transplantation. Therefore, this method can be used for large segment bone exposure.

## 1. Introduction

Lower limb fracture is a common traumatic injury often referred to orthopedic surgeons. Bones tend to fracture with different degrees of soft tissue damage. It is estimated that in the soft tissue injury category, the traumatic orthopedic injury ratio is as high as 82.8%.^[1]^ Violation due to weak tibial anterior soft tissue trauma and infection cause varying degrees of pretibial soft tissue defects, and during the process of open injury treatment, thorough debridement of soft tissue and bone will aggravate the degree of soft tissue defects. Failure to adequately manage soft tissue injuries can lead to serious complications such as bone exposure, osteonecrosis, increased infection, and the possibility of amputation. Further, the time necessary to process effective soft tissue coverage is critical to managing complications that can lead to potential infections and unnecessary amputations.^[2]^ Existing literature shows that drilling open a window on the exposed bone helps in the vascularization of the tissue and can promote the growth of granulation tissue.^[3]^ The continuous development of medical advances has enabled innovation in wound repair and reconstruction methods. These include various skin flaps from the initial graft, improved cure rate of wounds, shortened treatment time, and reduced amputation rates. However, in the face of large soft tissue defects and long bone exposed wounds, application of the flap technique can present major challenges.

## 2. Case report

**Figure 1.**
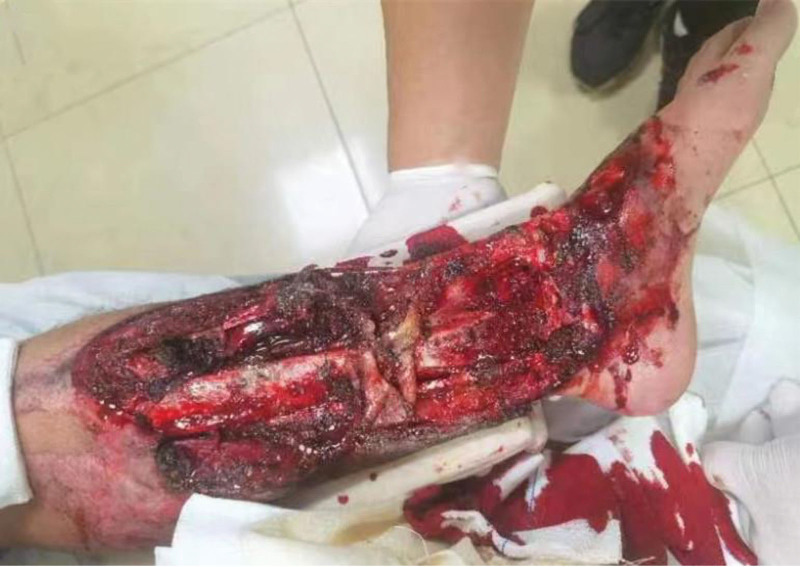
The appearance of the wound at the time of injury.

A 28-year-old healthy man sustained injury to the left lower leg due to a traffic accident. He was admitted to the local hospital and received a DR/CT examination (Fig. 1). The patient suffered a left ankle fracture, clavicle fracture and dislocation. He underwent debridement followed by Vacuum Sealing Drainage (VSD) cover and fibula plate fixation surgery. He was moved to the ICU postoperatively. One week later, he underwent open reduction and internal fixation of clavicle fracture surgery. Postoperative healing of the left shoulder wound was satisfactory, but the left lower leg showed poor wound healing and a large-area skin defect, and the long bone remained exposed. The patient returned to our hospital for further treatment.

### 2.1. Physical examination

The patient presented with stable vital signs and absence of any cardiopulmonary abnormalities; the wound covering the VSD had good negative pressure. There was a weak dorsalis pedis artery, toes showed restricted movement, the shoulder wound healed completely, dorsalis pedis pulse improved, the posterior tibial artery pulse remained unchanged, and the lateral margins of the left foot felt numb. General physical examination results were without exception.

### 2.2. Laboratory examination

The blood test results of the patient showed white blood cell count of 12.36 * 109/ L, neutrophil percentage of 80.3 %, C-reactive protein of 53.66 mg/L, and calcitonin of 0.115 (0–0.05 ng/mL).

### 2.3. Diagnose

Severe open wound to the left calf, Large skin defect on left calf, Extensive bone exposure in the left calf, Left medial malleolar defect Positive after admission to cephalosporins anti-infection treatment, 7 Ye Zao nucleoside detumescence, low molecular heparin calcium sodium anticoagulation. Four days after admission and debridement surgery, the patient had a large left leg skin defect, the tibia was exposed (area: 23 cm * 10 cm), the ankle area had defects, and the medial malleolus osseous was absent (Fig. 2). Ankle joint debridement take part of the training for the inflammatory tissue + drug susceptibility, after thorough debridement, will narrow rear to lift ahead of soft tissue wound, finally use the VSD cover the wound. After 4 days of germiculture, results revealed the presence of enterococcus, and the patient received levofloxacin to fight infection. After 7 days, the VSD was dismantled, the wound and exposed bone area had become smaller, and the surrounding granulation tissue was fresh. The medication of the patient was changed (Fig. 3). After 14 days, the patient underwent a second surgery, where intraoperative debridement was carried out on the wound again; ipsilateral thigh skin transplantation was undertaken and covered with VSD. The skin graft grew well, leaving the long bone exposed (Fig. 4). After 4 weeks, the skin graft area was observed to be healing well. The exposed bone was ground intraoperatively and subjected to micropower drilling such that each hole was spaced 5 to 10 mm apart (Fig. 5). Gentamicin bone cement (DuPuy Synthes, USA) was mixed with powder bone cement (40 g); polymethyl methacrylate reagent (DuPuy Synthes, USA) was added and fully mixed. The cement dough was placed to cover the bone shape and fixed with sutures (Fig. 6). The bone cement was removed, and the tissue showed no signs of necrosis (Fig. 7). Using an external fixator with the fibula plate, the fibula segment and trim were removed. The fibula ankle bone graft showed fusion and wound healing. The patient was followed up 1 month after discharge, and the affected area showed no infection, indicating recovery (Figs. 8–10).

**Figure 2-10.**
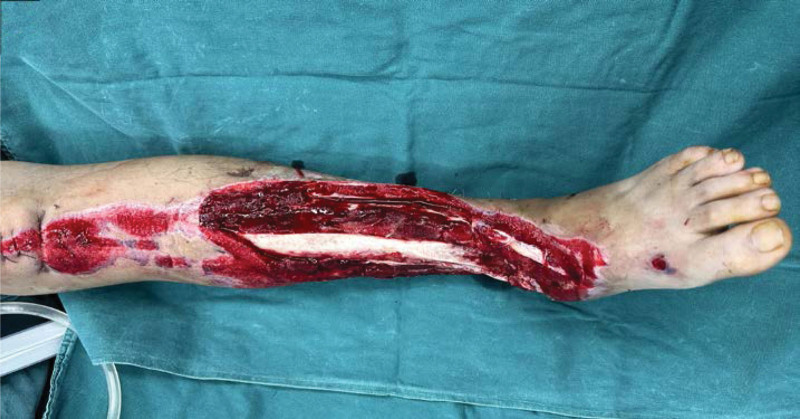
Treatment effects at each stage of the patient’s admission. It can be seen that the wound gradually shrinks, the exposed bone grows fresh granulation and induction membrane after drilling and covering with bone cement, and finally the ankle joint is fused, the wound is completely closed without any signs of infection or necrosis.

**Figure 3.**
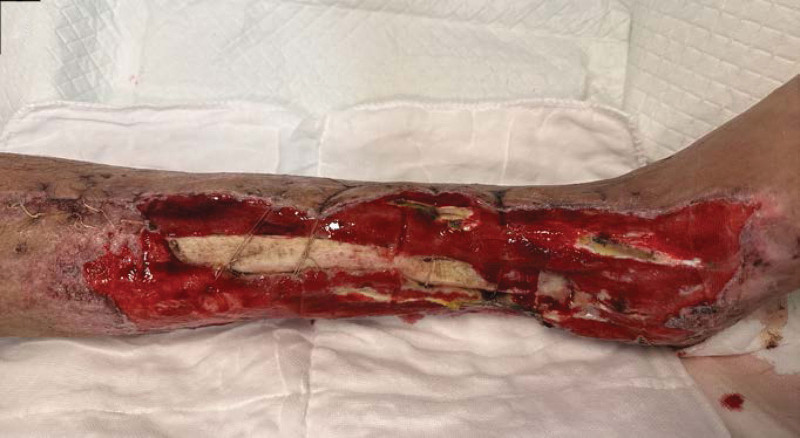


**Figure 4.**
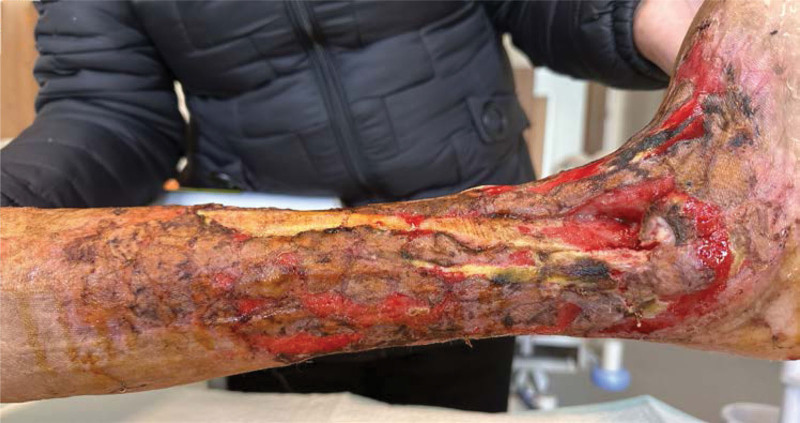


**Figure 5.**
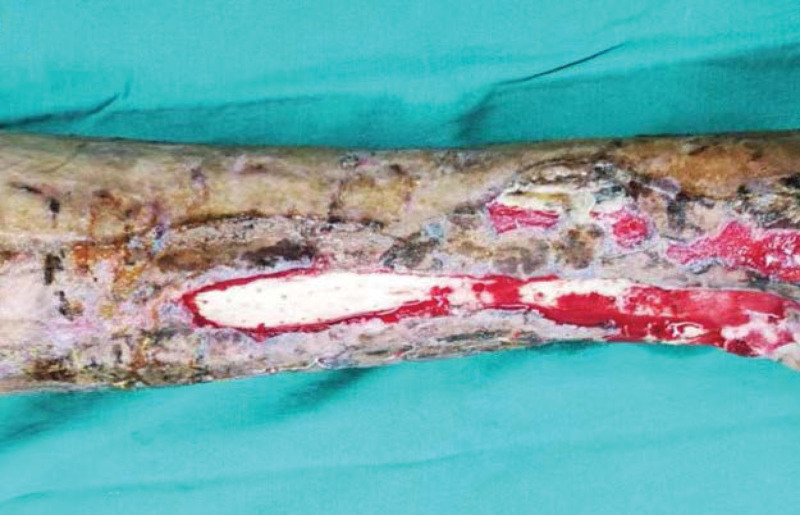


**Figure 6.**
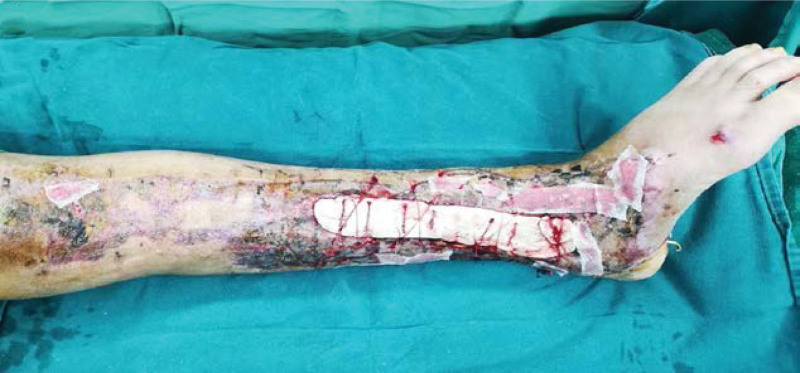


**Figure 7.**
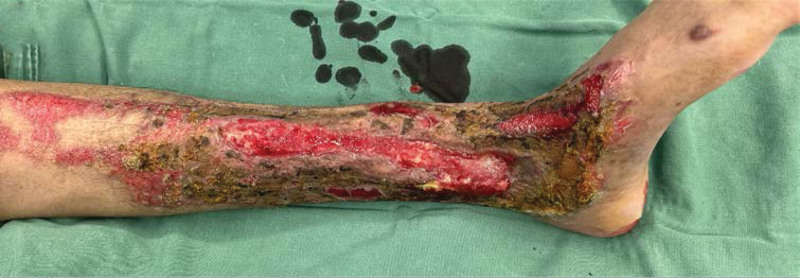


**Figure 8.**
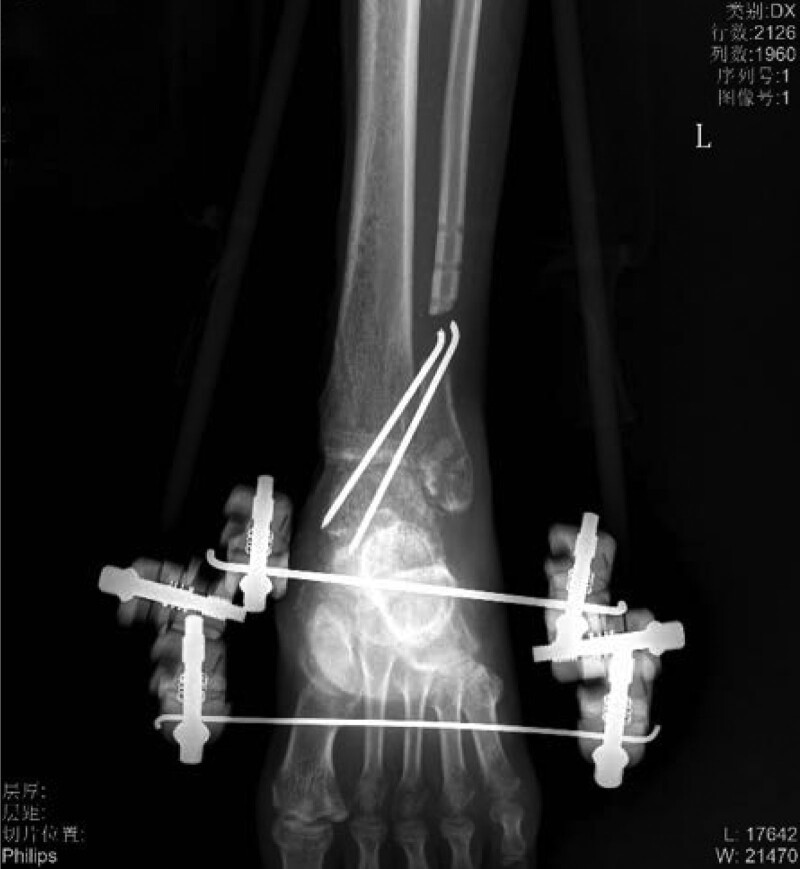


**Figure 2-10.**
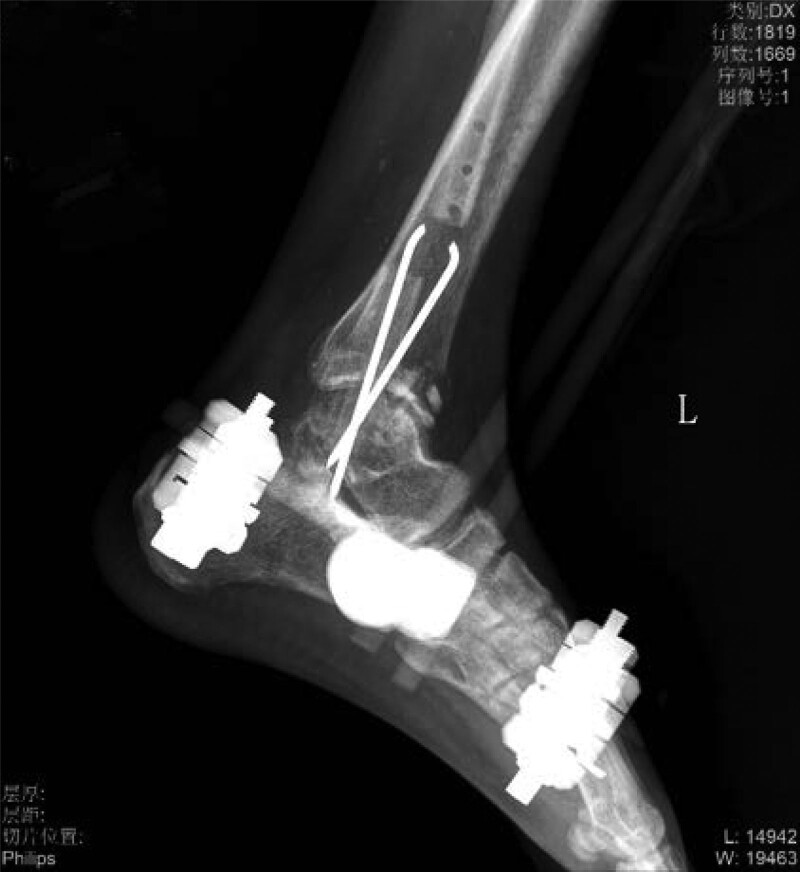
Continued

**Figure 10.**
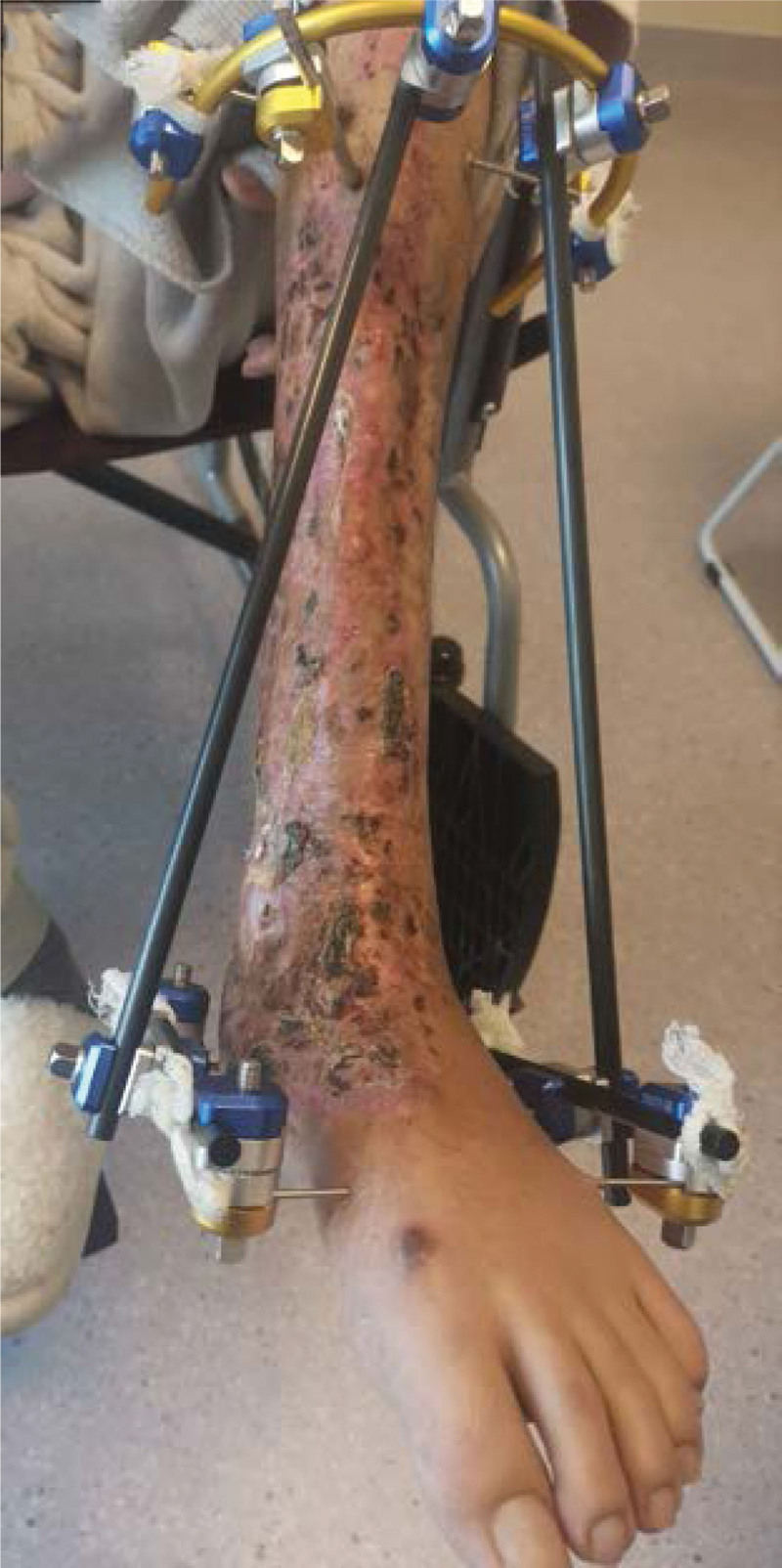


## 3. Discussion

Calf injury, both superficially and within the soft tissue, post suturing large-area skin lesions is not clinically rare; often, it occurs due to high-speed collisions, such as mechanical or traffic accident injury. Injury to the lower leg can result in a large section of the shin bone becoming exposed, often with bone defect and osteomyelitis. The treatment occurs in 2 steps: complete debridement first, which may or may not lead to secondary infections, and bone and soft tissue healing. In the case reported here, the main problem in treatment lay in the medial crus skin soft tissue defect covering a large-area, as well as posterior tibial vascular rupture defect, long exposed tibia with periosteum stripping, and medial malleolus bone loss with ligament damage but with dry tibia integrity, no fracture, and no other defects. Local soft tissue blood supply and full coverage are important factors that affect the prognosis of the wound.

In the face of long bone exposure, local skin flap pedicle transfer is often limited, and a free flap is recognized as an effective method for the repair of large-area wounds.^[4]^ It is critical to choose the area of reconstruction of vascular pedicle flap so as to facilitate blood circulation.^[5]^ The incoming and outgoing anterior and posterior tibial vessels are crucial vessels in the calf. When patients generally suffer from severe open injury or disfigurement of the lower leg, only 1 major blood vessel is unobstructed, and the other vessels suffer a knock-on effect of the local injury. They lose function due to the vessel pedicle being in the receiving area as a result of severe trauma. Even if possible, the operation of the flap is challenging. Some flaps affect appearance and require secondary repair to restore function. Treatment in our patient was challenging because of medial ankle injury and posterior tibial blood tube rupture; the large-area of tissue flap transfer presented a bigger risk and a long observation period. Therefore, we chose the cortex drilling Masquelet technology for wound repair.

As early as 500 Before Christ, Hippocrates^[6]^ described a skull drilling process and pointed out that to avoid thermal damage to the living bone, cooling water can reduce the local temperature. In 2011, Liu et al,^[7]^ with the help of gram drilling needle and closed negative pressure, increased the haversian bone plate in the trabecular bone through a pipe where a central pipe communicated with each other, promoting the growth of granulation tissue. In our patient, the damage to the surface of the cortical tibial periosteum and microvasculature was serious and led to different degrees of bare bone cortex necrosis. However, the surface of the bone cortex deep trabecular bone had not been impacted, and the haversian system was intact. Therefore, we used a micropower drill to remove necrotic bone and bore a hole until internal bleeding ensued (not drilling through medullary cavity). Studies have shown that increasing local blood flow induces cell proliferation and promotes wound healing.^[8]^ Liu^[7]^ used drilling after using the VSD, which closed the wound; however, we sliced with the bone cement cover directly on the surface of exposed bone, enabling surface-induced membrane formation. This technology is also called the Masquelet technique (induced membrane technique). The Masquelet technique was first reported in 1986 by French scholars for the treatment of large bone defects^[9]^; it is based on human foreign body reaction and bone regeneration.

In recent years, the use of gentamicin bone cement in the treatment of various types of trauma has shown some success.^[10]^ When in contact with body tissues, bone cement causes a foreign body reaction, thus forming an induced membrane. Masquelet induced synovial membrane thickening of 0.5 to 2.0 mm, and induced membranes carry out angiogenesis.^[11]^ Immune organizational learning analysis shows that the induced membrane capsule is composed of type I collagen, its inner layer is composed of the epithelial cells of the synovial membrane, and its outer layer is rich in fibroblasts. Simultaneously, the induced membrane capsule not only distributes small blood vessels in the long axis direction but can also secrete vascular endothelial growth factor, transforming growth factor-beta 1, and bone morphogenetic protein-2 to promote local blood vessel formation.^[12,13]^ However, no cases have been reported of direct coating of the bone with antibiotic-containing bone cement to form fresh induced membranes. In our patient, after the drilling, bone cement covered the bone surface. The advantage of this lies in the fact that, on the 1 hand, it can stimulate the body to produce a better foreign body reaction. On the other hand, it isolates the healing bone from the outside world, preventing bacteria from entering. Fresh induction membranes were formed on the bone surface by cortical drilling with the Masquelet technique, and no further skin grafting was performed. Considering the serious trauma of the patient, the regrafting of skin will cause new damage, whether the smooth healing after skin grafting and other factors. The wound in our case was only 19 cm on the long axis and 4 cm on the transverse axis. Therefore, we decisively chose palliative treatment (namely, healing through skin crawling instead). Care should be taken to not attach the auxiliary materials directly to the wound during dressing change to avoid wound damage when the gauze is taken out for a dressing change again as this prolongs the healing cycle. We only use cotton pads and loose bandages for external wrapping, thus ensuring that the skin crawling path is unimpeded, leading to shortened closing time.

In medial malleolus defects and ligament defects, we employ ankle fusion. Before surgery, we considered that medial malleolus and ligament injuries seriously affected ankle joint stability. Moreover, the psychological tolerance of patients after multiple operations decreases markedly. Finally, the treatment ended at the expense of ankle function. Reports of traumatic medial malleolus defect merger cases and medial collateral ligament injuries are few and mostly as case reports. Abbo et al^[14]^ reported a case of 11 children with medial malleolus defect after trauma and serious ligament damage who were treated using autogenous iliac (with hip fascia) and flap reconstruction of the medial malleolus; the short-term follow-up outcome was good. However, the lack of long-term results and small number of cases make it difficult to assess this technology effectively. Even with the progress in artificial ankle joint technology, ankle fusion and ankle reconstruction are still challenging. Morton Murdock – for the first time in 1970 – described the clinical use of an artificial ankle.^[15]^ Regarding ankle replacement indication, factors like patient age, type of injury (traumatic vs nontraumatic injury), sex, and presence of arthritis should be considered to choose the most suitable candidate. Kofoed^[16]^ pointed out that age < 50 years should be considered for artificial ankle joint replacement or similar curative effect. Most scholars believe that ankle replacement indications include age < 50 years old, of light weight, and little physical activity. In such situations, a conservative approach is preferred. A partial stabilization effect is achieved through scar healing. Artificial ankle arthroplasty can be performed even if severe ankle arthritis develops later.

## 4. Conclusion

Through the diagnosis and treatment of this case, we believe that drilling combined with bone cement technique is effective in the treatment of large exposed bones and avoids the free tissue flap coverage. The combination of drilling bleeding and bone cement foreign body reaction stimulates and induces membrane formation, which is a good foundation for treatment. In future treatments, this method can be used. However, the premise is to ensure that the bone trabeculae in the deep side of the bone cortex has not been significantly affected and the Havers system has not been destroyed. However, this is just an individual case, and there is still a lack of analysis of large samples.

## Author contributions

**Data curation:** Yuan Pan, Congtao Wang, Maohua Tang.

**Investigation:** Zhijun Dong.

**Resources:** Min An.

**Writing – review & editing:** Yingying Deng, Fuyao Liu.
